# Post‐epidemic investigation of Schmallenberg virus in wild ruminants in Slovenia

**DOI:** 10.1111/tbed.13495

**Published:** 2020-02-07

**Authors:** Gorazd Vengušt, Diana Žele Vengušt, Ivan Toplak, Danijela Rihtarič, Urška Kuhar

**Affiliations:** ^1^ Institute of Pathology, Wild Animals, Fish and Bees Veterinary Faculty University of Ljubljana Ljubljana Slovenia; ^2^ Institute of Microbiology and Parasitology Veterinary Faculty University of Ljubljana Ljubljana Slovenia

**Keywords:** ELISA, real‐time RT‐PCR, SBV, Schmallenberg virus, serology, wild ruminants

## Abstract

Schmallenberg virus (SBV) is a vector‐borne virus belonging to the genus *Orthobunyavirus* within the *Bunyaviridae* family. SBV emerged in Europe in 2011 and was characterized by epidemics of abortions, stillbirths and congenital malformations in domestic ruminants. The first evidence of SBV infection in Slovenia was from an ELISA‐positive sample from a cow collected in August 2012; clinical manifestations of SBV disease in sheep and cattle were observed in 2013, with SBV RNA detected in samples collected from a total of 28 herds. A potential re‐emergence of SBV in Europe is predicted to occur when population‐level immunity declines. SBV is also capable of infecting several wild ruminant species, although clinical disease has not yet been described in these species. Data on SBV‐positive wild ruminants suggest that these species might be possible sources for the re‐emergence of SBV. The aim of this study was to investigate whether SBV was circulating among wild ruminants in Slovenia and whether these species can act as a virus reservoir. A total of 281 blood and spleen samples from wild ruminants, including roe deer, red deer, chamois and European mouflon, were collected during the 2017–2018 hunting season. Serum samples were tested for antibodies against SBV by ELISA; the overall seroprevalence was 18.1%. Seropositive samples were reported from all over the country in examined animal species from 1 to 15 years of age. Spleen samples from the seropositive animals and serum samples from the seronegative animals were tested for the presence of SBV RNA using real‐time RT‐PCR; all the samples tested negative. Based on the results of the seropositive animals, it was demonstrated that SBV was circulating in wild ruminant populations in Slovenia even after the epidemic, as almost half (23/51) of the seropositive animals were 1 or 2 years old.

## 1 INTRODUCTION

In late 2011, unspecific clinical symptoms were observed in dairy cattle in the town of Schmallenberg in the state of North Rhine‐Westphalia, near the Dutch/German border region (Hoffmann et al., 2012). Using specific RT‐qPCR, an orthobunyavirus of the Simbu serogroup was identified as the causative agent and named ‘Schmallenberg virus’ (SBV). SBV's RNA genome is phylogenetically close to those of Shamonda virus, Aino virus and Akabane virus (Garigliany et al., 2012; Hoffmann et al., 2012). *Culicoides* biting midges play an essential role in the transmission of SBV, and they most likely spread the infection in many European countries (De Regge et al., 2014). SBV infections in adult ruminants are generally asymptomatic or may produce only mild unspecific signs, such as fever, diarrhoea and reduced milk production. When SBV‐naive dams are infected during a critical period of pregnancy, the infection can cause premature birth or stillbirth with severe foetal malformation (Bayrou et al., 2014; Hoffmann et al., 2012; Wernike, Hoffmann, et al., 2013).

Since its emergence, SBV spread rapidly among European livestock from the initial area of detection (Afonso et al., 2014). In 2013, SBV was first identified in Slovenia in a flock of 23 sheep in which nine aborted foetuses with malformations were observed on a farm. Between January and April 2013, SBV RNA was detected by real‐time RT‐PCR in samples collected from a total of 28 herds in which clinical manifestations of SBV disease in sheep and cattle were observed. Additionally, two archived samples collected in September 2012 were identified as SBV‐positive, confirming that SBV infection was already present in Slovenia in 2012 (Toplak, Cociancich, Rihtarič, Juntes, & Paller, 2014).

Schmallenberg virus is also capable of infecting several wild ruminant species, and early and quick spread of SBV has been observed, although clinical disease has not yet been described in these species (Laloy et al., 2014; Rossi et al., 2017). Thus, most of the published data regarding SBV infections in wildlife are based on the detection of antibodies in serum samples collected from animals without clinical signs characteristic of SBV infection. Regarding wild ruminants, SBV‐specific antibodies have been detected in deer, European mouflon, European bison, elk, chamois, Alpine ibex and moose (Chiari et al., 2014; Garcia‐Bocanegra et al., 2017; Laloy et al., 2014; Larska, Krzysiak, Kesik‐Maliszewska, & Rola, 2014; Larska, Krzysiak, Smreczak, Polak, & Zmudzinski, 2013; Linden et al., 2012; Malmsten et al., 2017; Mouchantat et al., 2015; Rossi et al., 2017), and SBV RNA was detected in two red and one fallow deer in Spain (Garcia‐Bocanegra et al., 2017). According to the detection of SBV antibodies in wild ruminants, these species might play a role in the epidemiology of SBV (Garcia‐Bocanegra et al., 2017; Larska et al., 2014). Wild ruminants may increase the risk of spillover transmission to livestock, especially in regions where they frequently share the same habitats (Rossi et al., 2017). In areas where conditions are favourable for the vectors and where wild ruminants can act as a reservoir, the virus may also become endemic (Garcia‐Bocanegra et al., 2017).

The aim of this study was to investigate whether SBV was or is circulating among wild ruminants in Slovenia and whether these species can act as a potential virus source in the re‐emergence of SBV.

## 2 MATERIALS AND METHODS

Samples from a total of 281 apparently healthy adult free‐range wild ruminants were collected throughout the country during the 2017/2018 hunting season (May 2017 to May 2018). Game wardens and hunters were encouraged to submit samples from animals shot during the regular annual cull. No ethical/welfare authority approval was required as all samples were collected post‐mortem. Prior to sampling, the hunters were instructed regarding the procedures and equipped with field sampling kits. Spleen and blood samples were collected from each carcass. Immediately after death, the blood samples were collected from the jugular vein or the heart. Samples were collected from 129 roe deer, 113 red deer, 29 chamois and 10 European mouflons of both sexes and various ages. Age was estimated subsequently by authorized committee of hunters during obligatory annual verification of hunted ungulates (Flajsman, Jerina, & Pokorny, 2017). Eruption patterns and tooth wear were used for age estimation of deer, whereas in chamois and mouflon, a horn growth rings method was used.

The animals were classified into two age groups: yearlings (<1 year old) and adults (>1 year old). Most samples (*n* = 223) were obtained from animals older than one year, namely, from 1 to 18 years of age, with a median age of 2 years. After sampling, the blood and spleen samples were transported to the Veterinary Faculty, University of Ljubljana. The blood samples were centrifuged for 10 min at 1,200 × *g* and stored in a −80°C freezer until use. The spleen samples were stored in a −80°C freezer until analysis.

### 2.1 ELISA

For SBV antibody detection in the serum samples, a commercial ELISA kit (ID Screen Schmallenberg Virus Indirect Screening Test Multi‐Species, ID. vet Innovative Diagnostics, France) was used according to the manufacturer's instructions. The results were interpreted according to the manufacturer's instructions.

### 2.2 Real‐time RT‐PCR

Serum samples from seronegative animals (*n* = 230) and spleen samples from seropositive animals (*n* = 51) were used for SBV RNA detection. Pools of 5 serum samples and 10% spleen suspensions in RPMI‐1640 (Gibco, Invitrogen Corporation) were prepared. Total RNA was extracted from 140 µl of spleen suspensions/serum sample pools using a QIAamp^®^ Viral RNA Mini Kit (Qiagen) according to the manufacturer's instructions. Previously designed primers, probes and a protocol for the detection of the SBV genome (L‐segment) by real‐time RT‐PCR method were used (Fischer et al., 2013; Toplak et al., 2014). The forward primer sequence SBV‐L1‐11F was 5′‐TTGCCGTTTGATTTTGAAGTTGTG‐3′, and the reverse primer sequence SBV‐L1‐155R was 5′‐TCAGGGATCGCAAATTAAAGAACC‐3′. The TaqMan probe sequence SBV‐L1‐36 was 5′‐FAM‐TCATCCGTGCTGACCCTCTGCGAG‐BHQ1‐3′. The real‐time RT‐PCR reaction included a reverse transcription step at 55°C for 10 min, followed by a denaturation step at 95°C for 10 min, and 45 cycles of 95°C for 15 s, 56°C for 20 s and 72°C for 30 s. To ensure the accurate performance of the real‐time RT‐PCR test, at least one positive control (SBV RNA provided by FLI; dilution of standard with a cycle threshold [Ct] of approximately 30) and one negative control (tissue sample of bovine origin from year 2010, free of SBV RNA) were included in each run.

### 2.3 Statistical analysis

The probability (and confidence intervals (CI); *p* = .05) of prevalence of antibodies against SBV (seroprevalence) was estimated taking into account the binominal distribution of data (antibodies detected, non‐detected) for each of the species × tests separately. The predicted number of seropositive animals for the population in the entire country was recalculated from sample to the entire population. Estimates of the population size for the study species were extracted from a professional report (Jerina et al., 2010) that applied age‐at‐harvest and pellet group counting method to estimate abundancy of all species of ungulates in the entire country. The data and methods used to estimate abundancy of wild ungulates were previously described (Klopčič, Jerina, & Bončina, 2010; Krofel et al., 2014).

## 3 RESULTS

**Figure 1:**
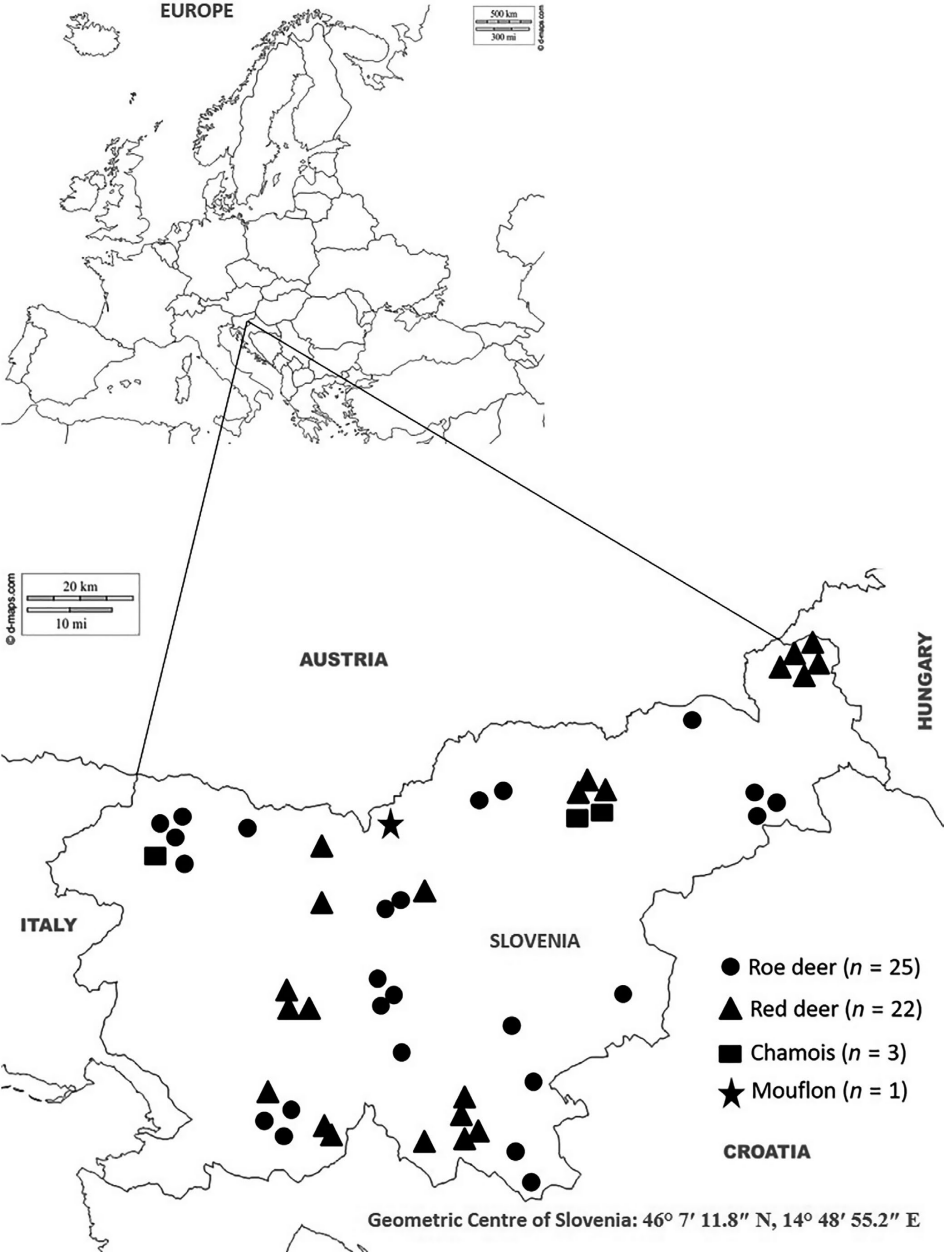
Geographical locations of SBV antibody‐positive samples of wild ruminants detected by ELISA

Based on the ELISA results, of the 281 serum samples, 51 (18.1%) were considered positive, and 230 (81.9%) were considered negative for the presence of SBV antibodies (Table 1). Seropositivity was detected in all the examined animal species, with the seroprevalence ranging from 10% in mouflon to 19.5% in roe deer. Detailed information regarding seroprevalence is presented in Table 3. Of the 51 positive samples, 50 samples were from adult animals from 1 to 15 years of age (Table 2). The geographical locations where positive animals were detected are shown in Figure 1.

**Table 1:** Results of the detection of specific antibodies against SBV using ELISA and the results of SBV RNA detection in spleen and serum samples from Slovenian wild ruminants using real‐time RT‐PCR

Species (No. and sex; median age)	ELISA	Real‐time RT‐PCR	
	Serum	Spleen	Serum
	No. positive (%)/No. tested	No. positive/No. tested	No. positive/No. tested
Roe deer (51 ♂, 78 ♀; 2)	25 (19.4)/129	0/25	0/104
Red deer (40 ♂, 73 ♀; 3)	22 (19.5)/113	0/22	0/91
Chamois (16 ♂, 13 ♀; 2)	3 (10.3)/29	0/3	0/26
Mouflon (3 ♂, 7 ♀; 2)	1 (10)/10	0/1	0/9
Total	51 (18.1)/281	0/51	0/230

**Table 2:** Data from the seropositive animals

Sample No.	Animal species	Sex	Age in Years	Month/Year of sampling
339	Roe deer	♂	<1	November/2017
86	Roe deer	♀	1	September/2017
96	Roe deer	♂	1	September/2017
209	Roe deer	♂	1	November/2017
109	Roe deer	♀	2	September/2017
110	Roe deer	♀	2	September/2017
196	Roe deer	♀	2	November/2017
202	Roe deer	♀	2	November/2017
212	Roe deer	♀	2	November/2017
329	Roe deer	♀	2	December/2017
356	Roe deer	♀	2	January/2018
93	Roe deer	♂	2	September/2017
133	Roe deer	♂	2	October/2017
366	Roe deer	♂	2	January/2018
328	Roe deer	♀	3	December/2017
365	Roe deer	♀	3	January/2018
76	Roe deer	♀	4	September/2017
1	Roe deer	♂	4	June/2017
113	Roe deer	♀	5	September/2017
118	Roe deer	♀	5	October/2017
288	Roe deer	♀	5	December/2017
330	Roe deer	♂	5	May/2017
283	Roe deer	♀	7	December/2017
332	Roe deer	♀	7	January/2018
254	Roe deer	♀	12	November/2017
137	Red deer	♀	1	October/2017
257	Red deer	♀	1	November/2017
138	Red deer	♂	1	October/2017
151	Red deer	♂	1	October/2017
168	Red deer	♀	2	November/2017
216	Red deer	♀	2	November/2017
311	Red deer	♀	2	December/2017
127	Red deer	♂	2	October/2017
233	Red deer	♀	3	November/2017
240	Red deer	♀	3	November/2017
152	Red deer	♀	4	October/2017
253	Red deer	♀	4	November/2017
136	Red deer	♂	4	October/2017
266	Red deer	♀	5	November/2017
102	Red deer	♂	5	September/2017
103	Red deer	♂	6	September/2017
364	Red deer	♀	7	January/2018
264	Red deer	♀	8	November/2017
252	Red deer	♂	8	November/2017
220	Red deer	♀	12	November/2017
274	Red deer	♀	12	November/2017
246	Red deer	♀	15	November/2017
81	Chamois	♂	2	September/2017
145	Chamois	♂	3	October/2017
36	Chamois	♂	4	August/2017
131	Mouflon	♀	2	October/2017

The estimated population size of red deer, roe deer, chamois and mouflon in Slovenia is 20,000, 110,000, 11,000 and 1,500, respectively. The predicted number of seropositive animals in the entire country, which was calculated from sample to entire population, was 3,900, 21,000, 1,100 and 150 for red deer, roe deer, chamois and mouflon, respectively, with more detailed information presented in Table 3.

**Table 3:** SBV seroprevalence in wild ruminants and predicted number of SBV seropositive animals in the entire country

	No. of tested samples	% of seropositive (*)	Population size estimate (entire country)	Predicted number of seropositive animals in entire country (*)
Red deer	129	19.4 (12.6–26.2)	20,000	3,900 (2,500–5,200)
Roe deer	113	19.5 (12.2–26.8)	110,000	21,000 (13,000–29,000)
Chamois	29	10.3 (0.0–21.4)	11,000	1,100 (0–2,400)
Mouflon	10	10.0 (0–28.6)	1,500	150 (0–430)
Total	281	18.1 (13.6–22.7)	142,500	26,600 (16,000–37,500)

* Low and high CI for *p* = .05.

In all the spleen and serum samples, the presence of SBV RNA using real‐time RT‐PCR was not detected (Table 1).

## 4 DISCUSSION

The first identification of SBV infection in Slovenia was recorded in 2013 by real‐time RT‐PCR in samples collected from a total of 28 herds in which clinical manifestations of SBV disease in sheep and cattle were observed (Toplak et al., 2014). However, the first evidence of SBV infection in Slovenia came from an ELISA‐positive sample from cattle collected in August 2012. This sample was tested during a survey to define the exact time of SBV introduction into the country. Forty‐two samples from cattle, collected from June to October 2012, were tested for the presence of SBV antibodies using ELISA. Later, 87 randomly selected serum samples from cattle collected between January and February 2013 were screened for SBV antibodies using ELISA, revealing a high prevalence of 82.8%. For the identification of the circulation of SBV in herds, a follow‐up study involving 170 samples from young stock cattle (between 7 and 13 months) collected between August and November 2014 was conducted for the presence of SBV antibodies using ELISA. Only 6.5% of the samples were positive, revealing a low level of circulation of SBV among young stock cattle in 2014 (Toplak, Starič, Cociancich, Rihtarič, & Paller, 2015).

This study is the first to investigate the exposure of wild ruminants to SBV in Slovenia. The study was conducted on hunting grounds across the territory of Slovenia. The results revealed an overall seroprevalence of 18.1% during the 2017/2018 hunting season, which indicated that SBV was circulating among wild ruminants, similar to results observed in other European countries such as Spain, Germany and Poland (Garcia‐Bocanegra et al., 2017; Kesik‐Maliszewska et al., 2018; Mouchantat et al., 2015). Our results suggest that wild ruminants were exposed to SBV in 2015 and 2016 or even later after the initial epidemic, as almost half (23/51) of the seropositive animals were 1 or 2 years old during the 2017/2018 hunting season. If we consider that SBV antibodies can be detected for at least 24 months (Claine, Coupeau, Wiggers, Muylkens, & Kirschvink, 2018; Elbers, Stockhofe‐Zurwieden, & van der Poel, 2014) or even up to 6 years (Wernike, Holsteg, Szillat, & Beer, 2018) post‐infection in naturally infected cattle, wild ruminants in Slovenia could be exposed to SBV at any time point after the start of epidemic, as the age of investigated positive animals was as high as 15 years. Although SBV RNA in the present study was not detected in the spleen and serum samples, our results are consistent with those of previous reports on SBV screening in the tissue of wild ruminants in Belgium and Poland (Kesik‐Maliszewska et al., 2018; Linden et al., 2012). Data regarding SBV RNA detection in tissue samples from wild ruminants are limited. There is one survey from Spain reporting the detection of SBV RNA in spleen samples from deer, but the prevalence (1.2%) of SBV was very low (Garcia‐Bocanegra et al., 2017). The most reliable explanation for this finding is that ruminants experience short‐term viremia during the first week after SBV infection (Van Der Poel et al., 2014; Wernike, Eschbaumer, et al., 2013; Wernike, Hoffmann, et al., 2013); thus, it is almost impossible to detect SBV in wild ruminants in a post‐epidemic survey.

Red and roe deer in Slovenia usually inhabit the lowland; mouflon habitats include steep, sunny, mountainous slopes near tree lines; and chamois inhabit extensive and continuous areas in the Alps. The seropositivity in red and roe deer from this study was almost 20%, while seropositivity in chamois and mouflon was half of that of deer. As in Italy and Spain (Chiari et al., 2014; Fernandez‐Aguilar et al., 2014), the seroprevalence in the present study was, on average, higher in lowland species than in highland wild ruminants. In contrast, Rossi et al. (2017) showed that the seroprevalence above 800 m occurred, on average, 1 year after domestic outbreaks, and sampling mountain wild ruminants in the same year may produce contradictory interpretations, possibly explaining the results from Italy and Spain. According to Toplak et al. (2015), the highest seroprevalence in domestic ruminants in Slovenia was observed in 2013; this is probably the result of exposure of the naive population to SBV infection during 2012, which was 5 years before the start of sampling among wild ruminants.

The majority of SBV‐positive samples (*n* = 50) in this study were collected from adult animals; only one roe deer was under one year of age. According to Elbers et al. (2014), maternal antibodies against SBV in calves can persist for 5–6 months, which may also be the case in the present study.

Wildlife infection can potentially occur in areas with livestock infection (Yon et al., 2019). The geographical locations of 28 herds of sheep and cattle with SBV‐positive results in 2013 (Toplak et al., 2014) coincide with the geographical locations of the ELISA‐positive wild ruminants in the present study, similar to results previously observed in Sweden (Malmsten et al., 2017). The results of this study suggest that SBV was circulating in Slovenia in 2015 and 2016, similar to other European countries such as Germany and Spain (Garcia‐Bocanegra et al., 2017; Mouchantat et al., 2015) but not Sweden due to a long vector‐free season (Malmsten et al., 2017).

There have been several reports of low‐level SBV circulation among ruminants following the Schmallenberg epidemic in Europe (Collins, Barrett, Doherty, McDonnell, & Mee, 2017; Larska, 2018; Stavrou, Daly, Maddison, Gough, & Tarlinton, 2017), resulting in a reduced seroprevalence and probably immunity (Meroc et al., 2015; Wernike, Holsteg, Sasserath, & Beer, 2015). The mechanism by which SBV infection persists from season to season (overwinters) remains unclear (European Food Safety Authority, 2014). Some data suggest that *Culicoides* biting midges play an essential role in the transmission of SBV, and SBV may overwinter in these vectors (Larska, Lechowski, Grochowska, & Zmudzinski, 2013). Similarly, data on SBV‐positive wild ruminants suggest that these species might play a role in the epidemiology of SBV (Garcia‐Bocanegra et al., 2017; Larska et al., 2014; Stavrou et al., 2017) and thus are a possible source for the re‐emergence of SBV. When the population‐level immunity declines and the population of immunologically naive animals increases, the re‐emergence of SBV is predicted (Collins, Barrett, Doherty, Larska, & Mee, 2016; Stavrou et al., 2017).

In conclusion, the present study revealed that SBV was circulating among wild ruminants in Slovenia after the epidemic and might still be circulating.

## CONFLICTS OF INTEREST

The authors declare no conflicts of interest.

## ETHICAL APPROVAL

No ethical/welfare authority approval was required, as all samples were collected post‐mortem.

## Data Availability

Research data are not shared.
